# A promoter polymorphism in APJ gene is significantly associated with blood pressure changes and hypertension risk in Chinese women

**DOI:** 10.18632/oncotarget.13370

**Published:** 2016-11-15

**Authors:** Guofeng Li, Xingyuan Sun, Dalong Zhao, Lan He, Lihong Zheng, Jing Xue, Bin Wang, Hongming Pan

**Affiliations:** ^1^ Department of Human Anatomy, School of Basic Medicine, Qiqihar Medical University, Qiqihar, Heilongjiang, China; ^2^ Department of Neurology, The Third Affiliated Hospital of Qiqihar Medical University, Qiqihar, Heilongjiang, China; ^3^ Clinical Laboratory, Qiqihar Jianhua Hospital, Qiqihar, Heilongjiang, China; ^4^ Department of Advanced Mathematics, School of Basic Medicine, Qiqihar Medical University, Qiqihar, Heilongjiang, China; ^5^ Department of Biogenetics, School of Basic Medicine, Qiqihar Medical University, Qiqihar, Heilongjiang, China; ^6^ Department of Immunology, School of Medical Technolog, Qiqihar Medical University, Qiqihar, Heilongjiang, China; ^7^ Department of Physiology, School of Basic Medicine, Qiqihar Medical University, Qiqihar, Heilongjiang, China; ^8^ Department of Biochemistry, School of Basic Medicine, Qiqihar Medical University, Qiqihar, Heilongjiang, China

**Keywords:** essential hypertension, blood pressure, apelin/APJ system, polymorphism, association

## Abstract

The aim of this study was to interrogate the gender-specific association of 5 well-defined polymorphisms in apelin/APJ system with both blood pressure changes and hypertension risk in a northeastern Chinese population. This is a population-based case-control study, including 650 hypertensive patients and 645 normotensive controls. Data were analyzed by STATA and Haplo.Stats. The genotype distributions of 5 study polymorphisms were in Hardy-Weinberg equilibrium in both genders. The rs7119375 and rs10501367 were completely linked. The genotypes (*P* = 0.001) and alleles (*P* < 0.001) of rs7119375 differed significantly between patients and controls in women. Carriers of rs7119375-AA genotype had significant higher systolic blood pressure (SBP) than carriers of rs7119375-GG genotype in both patients and controls of female gender (*P* < 0.01). Moreover, carriers of rs7119375-A allele were 1.80 times more likely to develop hypertension relative to carriers of rs7119375-GG genotype after adjusting for age, body mass index and glucose (odds ratio: 1.80; 95% confidence interval: 1.03–3.16; *P* = 0.040). Further allele combination analysis supported the leading contribution of rs7119375 to hypertension risk. Our findings demonstrated that the mutation of promoter polymorphism rs7119375 in APJ gene was significantly associated with elevated SBP and increased hypertension risk in Chinese women.

## INTRODUCTION

Hypertension is a complex polygenic disorder [1]. Many susceptible genes underlie its pathogenesis. Apelin is one of hypertension-susceptibility genes [2, 3]. Apelin plays a vital role in cardiovascular system through its 7-transmembrane-receptor, APJ [4, 5]. Apelin and APJ are highly expressed in vessel walls, especially in endothelial cells [6, 7]. There is strong evidence that apelin/APJ system is a promising therapeutic target for hypertension [8, 9]. The genes encoding apelin and APJ are polymorphic [10]. By direct sequencing, the initial family-based association study has identified 5 common polymorphisms in apelin/APJ system associated with hypertension [11]. This association was subsequently validated in a large case-control association study [12], while the relationship between these polymorphisms and blood pressure remains unresolved. As indicated by the GenSalt study, *apelin* and *APJ* genetic alterations may influence blood pressure response to both potassium intake [13] and dietary sodium intervention [14]. In light of these observations, it would be tempting to postulate that genetic mutation of apelin/APJ system can regulate blood pressure and result in the development of hypertension. In addition, apelin is a peptide hormone and its coded gene is mapped on X-chromosome. Growing genetic data on sex chromosomes emphasize the gender-specific role in the pathogenesis of hypertension [15, 16]. To shed more light on this issue, we decided to interrogate the gender-specific association of 5 well-defined polymorphisms in apelin/APJ system with both blood pressure changes and hypertension risk in a northeastern Chinese population.

## RESULTS

### Baseline characteristics

Table 1 compares the baseline characteristics of study group between genders. There were 322 and 364 men out of 650 hypertensive patients and 645 normotensive controls, respectively. In both genders, age and BMI were comparable between patients and controls, as well as for glucose and triglycerides. Patients had higher mean levels of total cholesterol and LDLC than controls in both genders (*P* ≤ 0.001). For HDLC, an elevated level was seen in controls relative to patients for women only (*P* = 0.005). In contrast, circulating levels of apolipoprotein A and B were significantly higher in patients than in controls for men only (*P* = 0.001 and 0.034, respectively).

**Table 1:** The baseline characteristics of study group by gender

Characteristics	Men			Women		
	Patients	Controls	*P*	Patients	Controls	*P*
	(*n* = 322)	(*n* = 364)		(*n* = 328)	(*n* = 281)	
Age (years)	61.16 (9.98)	61.12 (9.60)	0.850	65.31 (9.03)	64.50 (9.14)	0.271
BMI (kg/m^2^)	25.41 (3.31)	25.29 (3.02)	0.780	24.72 (3.40)	25.14 (3.48)	0.383
SBP (mmHg)	151.68 (14.64)	121.84 (9.83)	< 0.001	152.69 (13.66)	122.14 (9.68)	< 0.001
DBP (mmHg)	88.89 (11.51)	76.00 (8.67)	< 0.001	86.11 (10.70)	75.82 (8.06)	< 0.001
Glucose (mmol/L)	5.73 (1.84)	5.77 (1.76)	0.771	5.92 (1.91)	5.77 (1.80)	0.336
TG (mmol/L)	1.79 (0.91)	1.81 (1.01)	0.776	1.89 (1.13)	1.86 (0.92)	0.726
TC (mmol/L)	4.55 (1.09)	4.24 (0.88)	< 0.001	4.97 (1.07)	4.68 (1.12)	0.001
HDLC (mmol/L)	1.09 (0.26)	1.10 (0.35)	0.655	1.24 (0.29)	1.31 (0.32)	0.005
LDLC (mmol/L)	2.76 (0.95)	2.51 (0.70)	< 0.001	2.96 (0.92)	2.72 (0.85)	0.001
ApoA (mmol/L)	1.23 (0.24)	1.18 (0.20)	0.001	1.35 (0.29)	1.32 (0.26)	0.085
ApoB (mmol/L)	0.91 (0.24)	0.87 (0.22)	0.034	0.96 (0.28)	0.93 (0.40)	0.217

*Abbreviations*: BMI, body mass index; SBP, systolic blood pressure; DBP, diastolic blood pressure; TG, triglycerides; TC, total cholesterol; HDLC, high-density lipoprotein cholesterol; LDLC, low-density lipoprotein cholesterol; ApoA, apolipoprotein A; ApoB, apolipoprotein B. *P* was calculated by *t*-test or Mann-Whitney *U* test where appropriate.

### Single polymorphism analysis

**Figure 1:**
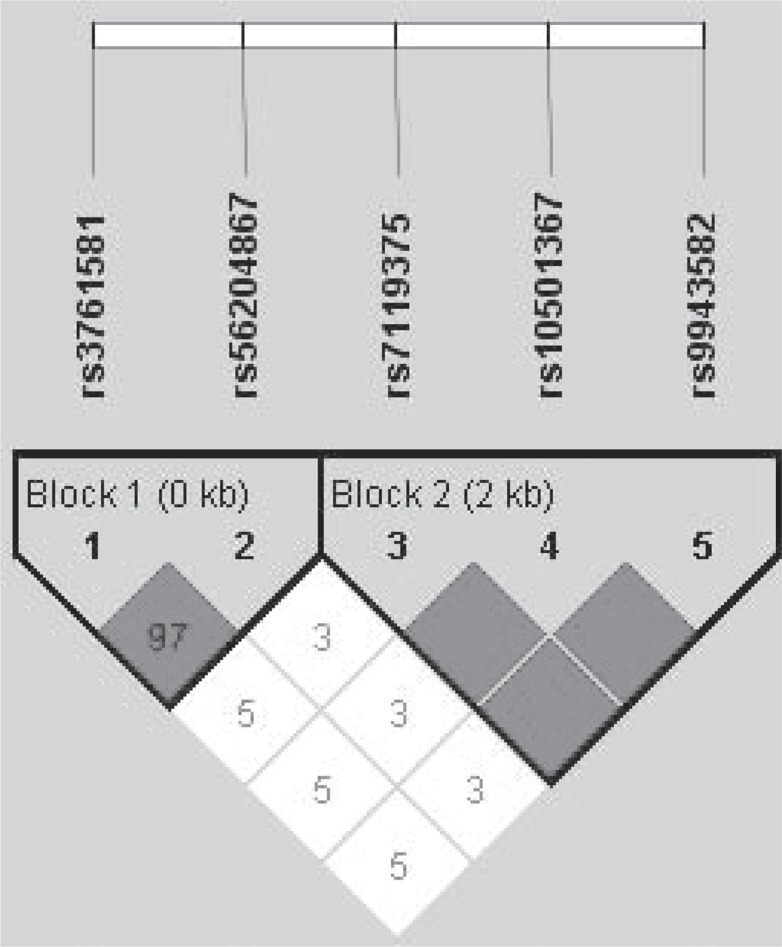
The linkage disequilibrium of 5 examined polymorphisms in apelin/APJ system

Linkage disequilibrium analysis showed that rs7119375 and rs10501367 were completely linked (Figure 1), and so only one polymorphism (rs7119375) was retained for analysis. Genotype distributions of the remaining 4 polymorphisms satisfied the Hardy-Weinberg expectations in both genders (*P* > 0.05). The genotype distributions and allele frequencies of 4 polymorphisms are presented by gender in Table 2. There were significant differences in the genotypes (*P* = 0.001) and alleles (*P* < 0.001) of rs7119375 in women, even after the Bonferroni correction (*P* < 0.05/5, here 5 is the total number of polymorphisms under study), and the power to detect this significance was over 95%. No significance was observed for the other genetic comparisons in both genders.

**Table 2:** The genetic distributions of 4 polymorphisms in apelin/APJ system by gender

Polymorphisms		Men			Women		
		Patients	Controls	*P*	Patients	Controls	*P*
rs3761581	TT	194 (60.25)	216 (59.34)		161 (49.09)	128 (45.55)	
	TG			0.809	128 (39.02)	117 (41.64)	0.684
	GG	128 (39.75)	148 (40.66)		39 (11.89)	36 (12.81)	
	G	39.75%	40.66%	0.732	31.40%	33.63%	0.408
rs56204867	AA	214 (66.46)	241 (66.21)		160 (48.78)	120 (42.70)	
	AG			0.945	131 (39.94)	129 (45.91)	0.289
	GG	108 (33.54)	123 (33.79)		37 (11.28)	32 (11.39)	
	G	33.54%	33.79%	0.922	31.25%	34.34%	0.251
rs7119375	GG	182 (56.52)	225 (61.81)		179 (54.57)	190 (67.62)	
	GA	126 (39.13)	124 (34.07)	0.362	130 (39.63)	87 (30.96)	0.001
	AA	14 (4.35)	15 (4.12)		19 (5.79)	4 (1.42)	
	A	23.91%	21.15%	0.222	25.61%	16.90%	<0.001
rs9943582	CC	179 (55.59)	222 (60.99)		175 (53.35)	161 (57.30)	
	CT	121 (37.58)	119 (32.69)	0.352	122 (37.20)	105 (37.37)	0.148
	TT	22 (6.83)	23 (6.32)		31 (9.45)	15 (5.34)	
	T	25.62%	22.66%	0.201	28.05%	24.02%	0.111

Genotype data are expressed as number (percentage). *P* was calculated by χ^2^ test or Fisher's exact test where appropriate.

The changes of SBP and DBP across genotypes of 4 examined polymorphisms are summarized in Table 3. For SBP, carriers of rs3761581-GG genotype had lower SBP than carriers of rs3761581-TT genotype in female controls (*P* < 0.05). In addition, carriers of rs7119375-AA genotype had significant higher SBP than carriers of rs7119375-GG genotype in both patients and controls of female gender (*P* < 0.01). No significant changes were identified in women for DBP, and in men for both SBP and DBP (*P* > 0.05).

**Table 3:** The changes of systolic and diastolic blood pressure across genotypes of 4 examined polymorphisms by gender

Polymorphisms		Men		Women	
		Patients	Controls	Patients	Controls
rs3761581	TT	151.61 (14.29)	122.07 (9.45)	153.81 (14.45)	125.33 (7.92)
	TG	NA	NA	151.69 (12.24)	122.71 (8.95)
	GG	151.78 (15.21)	121.68 (10.11)	151.38 (14.69)	120.52 (10.65)*
rs56204867	AA	152.61 (15.79)	121.82 (10.10)	153.71 (14.65)	122.53 (9.01)
	AG	NA	NA	151.73 (12.06)	122.16 (10.55)
	GG	151.21 (14.04)	121.88 (9.34)	151.73 (14.59)	124.59 (8.07)
rs7119375	GG	151.99 (13.69)	121.81 (9.92)	147.16 (14.45)	117.50 (15.00)
	GA	151.37 (15.08)	121.88 (9.72)	152.02 (12.99)	120.85 (9.49)
	AA	150.43 (22.17)	121.87 (10.15)	154.67 (14.00)*	123.97 (10.48)*
rs9943582	CC	149.59 (18.14)	120.09 (10.02)	148.39 (12.61)	123.13 (9.79)
	CT	151.63 (15.36)	121.93 (9.84)	152.19 (13.17)	122.58 (10.43)
	TT	151.97 (13.71)	122.00 (9.84)	153.81 (14.07)	122.75 (9.20)
**DBP**					
rs3761581	TT	89.30 (1167)	76.25 (8.73)	87.60 (12.14)	77.64 (7.12)
	TG	NA	NA	85.28 (11.43)	74.50 (7.84)
	GG	88.27 (11.27)	75.64 (8.59)	86.40 (9.70)	76.52 (8.36)
rs56204867	AA	89.11 (11.51)	76.17 (8.74)	85.38 (9.66)	76.37 (8.40)
	AG	NA	NA	85.50 (11.47)	74.74 (7.96)
	GG	88.46 (11.54)	75.67 (8.54)	87.07 (12.23)	78.13 (6.57)
rs7119375	GG	84.36 (11.36)	78.00 (8.62)	83.42 (9.87)	72.50 (9.57)
	GA	88.64 (11.56)	75.85 (8.57)	85.07 (10.91)	74.79 (7.81)
	AA	89.77 (11.17)	76.04 (8.90)	87.42 (10.65)	77.05 (8.58)
rs9943582	CC	85.95 (11.35)	76.70 (8.75)	83.55 (9.24)	74.00 (8.06)
	CT	88.63 (11.46)	75.84 (8.62)	86.40 (10.69)	75.90 (7.85)
	TT	89.81 (11.59)	76.18 (8.81)	86.34 (11.04)	75.96 (8.42)

*Abbreviations*: SBP, systolic blood pressure; DBP, diastolic blood pressure; NA, not available.

* *P* < 0.05.

The risk prediction of single polymorphisms for hypertension is provided in Table 4. Given the small number of mutant homozygous genotypes, the risk prediction was only calculated under additive and dominant models. The association of rs7119375 with hypertension was significant in women under both additive and dominant models, even after adjusting for age, BMI and glucose. For instance, carriers of rs7119375-A allele were 1.80 times more likely to have hypertension relative to carriers of rs7119375-GG genotype after confounding adjustment (OR = 1.80; 95% CI: 1.03–3.16; *P* = 0.040). No significance was detected for the other polymorphisms under both models.

**Table 4:** The risk prediction of 4 examined polymorphisms with hypertension under additive and dominant models by gender

Polymorphisms	Men		Women	
	OR; 95% CI;*P*	OR; 95% CI;P*	OR; 95% CI; *P*	OR; 95% CI;P*
*Additive model*				
rs3761581	0.98;0.84–1.14;0.809	0.98;0.75–1.29;0.907	0.91;0.72–1.15;0.426	1.03;0.70–1.53;0.867
rs56204867	0.99;0.85–1.17;0.945	0.94;0.71–1.25;0.671	0.87;0.69–1.11;0.259	1.01;0.68–1.51;0.953
rs7119375	1.18;0.91–1.53;0.211	1.40;0.89–2.22;0.148	1.75;1.31–2.35;<0.001	1.86;1.16–2.97;0.010
rs9943582	1.17;0.92–1.49;0.211	1.27;0.82–1.96;0.291	1.22;0.95–1.58;0.118	1.26;0.84–1.89;0.261
*Dominant model*				
rs3761581	0.96;0.71–1.31;0.809	0.97;0.56–1.68;0.907	0.87;0.63–1.19;0.384	1.08;0.63–1.86;0.778
rs56204867	0.99;0.72–1.36;0.945	0.89;0.51–1.55;0.671	0.78;0.57–1.08;0.134	0.96;0.56–1.65;0.879
rs7119375	1.25;0.92–1.69;0.159	1.43;0.83–2.47;0.198	1.74;1.25–2.42;0.001	1.80;1.03–3.16;0.040
rs9943582	1.25;0.92–1.69;0.152	1.32;0.77–2.27;0.315	1.17;0.85–1.62;0.330	1.23;0.72–2.13;0.448

*Abbreviations*: OR, odds ratio; 95% CI, 95% confidence interval.

* *P* was adjusted for age, body mass index and glucose.

### Allele combination analysis

Summarized in Table 5 are the comparison of derived allele combinations between patients and controls and their risk prediction for hypertension by gender. To avoid chance findings, only allele combination with a frequency > 3% in all participants is listed. In men, the most common allele combination was T-A-G-C (alleles in order of rs3761581, rs56204867, rs7119375 and rs9943582), which was frequency-matched between the two groups. Relative to this most common allele combination, the other allele combinations were not associated with hypertension risk. Contrastingly in women, the most common allele combination was T-A-G-C, and the allele combination T-A-A-T was significantly associated with 83% (OR = 1.83; 95% CI: 1.20–2.80; *P* = 0.005) and 84% (OR = 1.84; 95% CI: 1.20–2.81; *P* = 0.005) increased risk before and after adjusting for age, BMI and glucose, respectively. Another allele combination T-A-G-T had a higher frequency in controls than in patients (*P* = 0.001) and was in turn associated with 66% (OR = 0.34; 95% CI: 0.15–0.77; *P* = 0.010) and 67% (OR = 0.33; 95% CI: 0.15–0.76; *P* = 0.010) reduced risk for hypertension before and after confounding adjustment, respectively. After the Bonferroni correction, significance persisted for the allele combination T-A-A-T in women (*P* < 0.05/5, here 5 is the total number of estimated allele combinations).

**Table 5:** The allele combination analysis (with frequency > 3%) of 4 examined polymorphism and the risk prediction for hypertension by gender

Combination*	Patients	Controls	*P*	OR; 95% CI; *P*	OR; 95% CI; P**
**Men**					
T-A-G-C	45.03%	44.23%	0.807	Reference group	
G-G-G-C	24.38%	27.06%	0.394	0.90; 0.74–1.11; 0.345	0.90; 0.74–1.11; 0.344
T-A-A-T	13.98%	13.46%	0.815	1.05; 0.75–1.46; 0.787	1.05; 0.75–1.46; 0.785
G-G-A-T	7.92%	6.18%	0.254	1.29; 0.85–1.96; 0.227	1.29; 0.85–1.96; 0.225
G-A-G-C	4.50%	5.63%	0.509	0.84; 0.57–1.25; 0.391	0.84; 0.57–1.25; 0.397
**Women**					
T-A-G-C	48.44%	49.20%	0.888	Reference group	
G-G-G-C	22.75%	25.00%	0.251	0.91; 0.66–1.25; 0.560	0.91; 0.66–1.25; 0.549
T-A-A-T	18.30%	10.53%	< 0.001	1.83; 1.20–2.80; 0.005	1.84; 1.20–2.81; 0.005
G-G-A-T	6.96%	5.73%	0.124	1.39; 0.74–2.58; 0.304	1.40; 0.75–2.62; 0.288
T-A-G-T	1.24%	5.21%	0.001	0.34; 0.15–0.77; 0.010	0.33; 0.15–0.76; 0.010

*Abbreviations*: OR, odds ratio; 95% CI, 95% confidence interval.

* Alleles in order of rs3761581, rs56204867, rs7119375 and rs9943582.

** *P* was adjusted for age, body mass index and glucose.

## DISCUSSION

This is a replication study interrogating the association of five well-defined polymorphisms in apelin/APJ system with blood pressure changes and hypertension risk in a northeastern Chinese population. The key finding of this study was that the mutation of promoter polymorphism rs7119375, in complete linkage with rs10501367, in *APJ* gene was associated with elevated SBP and increased hypertension risk in Chinese women. This finding not only suggests a gender-specific role of apelin/APJ system in the pathogenesis of hypertension, but also provides an alternative therapeutic target for hypertension.

There is strong evidence that apelin/APJ system is implicated in the regulation of cardiovascular system [17]. Apelin is an endogenous ligand for the G protein-coupled receptor APJ, and they are widely distributed in central and peripheral tissues [18]. It is widely accepted that apelin/APJ system plays a crucial role in angiogenesis, a pathological hallmark of hypertension [19]. Also, this system can lower blood pressure likely through relaxing blood vessels and a nitric oxide-dependent mechanism [3]. Several investigators have observed a higher level of circulating apelin in hypertensive patients than in healthy controls [20], whereas others have not [2, 21]. A recent study found a significant inverse relation between circulating apelin and blood pressure [22]. There is wide recognition that circulating apelin changes are partly under genetic control. Given that the genes coding for apelin and APJ are polymorphic, it is crucial to identify genetic defects in apelin/APJ system that are responsible for blood pressure regulation and determine an individual's risk for developing hypertension. In this study, 5 polymorphisms selected were also located in the promoter regions, which might be biologically functional.

The *apelin* and *APJ* genes were sequenced by a previous pilot study by Li et al, and 12 common polymorphisms were identified and further genotyped in 1015 Han Chinese from 248 families [11]. Only 5 polymorphisms in apelin/APJ system were significantly associated with hypertension, obesity and onset age of hypertension. The results of this pilot study were subsequently validated by several association studies [10, 12, 15, 23]. However, significant findings are not often reproducible, and no consensus has been reached yet. To deepen our understanding on the genetic contribution of apelin/APJ system, we investigated the association of 5 well-defined polymorphisms with both blood pressure changes and hypertension risk among 650 hypertensive patients and 645 normotensive controls. In addition, we explored whether this association is gender-dependent. Via a comprehensive analysis, we found a predominant role of *APJ* gene rs7119375 polymorphism in regulating SBP and predisposing to hypertension in women, partly in line with the findings by Niu et al. [12], as the relationship between rs7119375 and blood pressure was not explored. By contrast in the GenSalt study, *APJ* gene rs7119375 polymorphism was not significantly associated with blood pressure response to potassium supplement [13]. In addition, our allele combination analysis confirmed the leading contribution of this polymorphism to hypertension predisposition. On the basis of these findings, we propose for the first time that the promoter polymorphism rs7119375 in *APJ* gene is a potential hypertension-susceptibility locus in women, and it might precipitate hypertension through regulating SBP.

Another important finding of this study was the gender-specific involvement of *APJ* gene in blood pressure regulation and hypertension predisposition. In fact, this gender-specific contribution may reflect the impact of some genes on sex chromosomes, such as apelin and angiotensin-converting enzyme 2 in X chromosome [24]. Several lines of evidence have suggested a heterogeneous contribution of these X-linked genes to the pathogenesis of hypertension [15, 25–27]. In spite of the null association of *apelin* gene with hypertension in this study, we observed that SBP differed significantly between two homozygous genotypes of rs3761581 in *apelin* gene. As with most mutations, the relative risk conferred by a single polymorphism might be small, and it will be worthwhile analyzing this polymorphism further in independent populations with different genetic backgrounds.

Several limitations should be acknowledged for this study. The first limitation is the case-control study design with study group retrospectively collected. The second limitation is that this is a population-based study, which cannot be extrapolated to the general population. The third limitation might be the insufficient statistical power arising from the limited sample size, especially by gender. The fourth limitation is that only five promoter polymorphisms from apelin/APJ system were genotyped. The fifth limitation is that circulating apelin levels are not measured at enrollment, and it is of interest to analyze the relationship between blood pressure and circulating apelin. The sixth limitation is that information on drug regimens, salt consumption and menopause is not available to exclude the residual confounding effects. The seventh limitation is our conclusion was based on only Chinese population and the application of our findings cannot be generalized to non-Chinese groups.

To sum up, our findings demonstrated that the mutation of promoter polymorphism rs7119375, in complete linkage with rs10501367, in *APJ* gene was associated with elevated SBP and increased hypertension risk in Chinese women. In addition, our findings also highlighted the gender-specific involvement of *APJ* gene in blood pressure regulation and hypertension predisposition. Pending future successful validation, our findings may provide the basis for further personalized medicine, namely, individuals with mutant *APJ* gene could be identified early and treated with optimal regimens since they could carry a high hypertension risk.

## MATERIALS AND METHODS

### Study group

The study group consisted of patients with essential hypertension and normotensive controls. In brief, they are unrelated local residents of Han nationality in Qiqihar city, Heilongjiang province, China. All study participants were enrolled between September 2013 and October 2015 from Qiqihar city and surrounding areas. Normotensive controls were enrolled from the participants who took part in the annual routine medical examinations at Qiqihar Jianhua Hospital and The Third Affiliated Hospital of Qiqihar Medical University. Each participant provided a written informed consent prior to participation. This study was approved by the institutional review boards at Qiqihar Medical University.

### Diagnosis and sample size

Blood pressure was measured three times with a mercury sphygmomanometer after the participants had rested for at least 5 minutes in the sitting position. The mean level of last two measurements was used for analysis. Hypertension is diagnosed as mean systolic blood pressure (SBP) ≥ 140 mmHg or mean diastolic blood pressure (DBP) ≥ 90 mmHg or receipt of antihypertensive regimens. Normal blood pressure is defined as mean SBP < 140 mmHg and mean DBP < 90 mmHg. Patients were excluded if they were clinically identified with secondary hypertension, coronary artery disease and renal related disorders. This study group included 650 hypertensive patients and 645 normotensive controls.

### Demographic and clinical indexes

Age, body weight and height were taken down at the time of enrollment. Body mass index (BMI) was calculated as weight in kilograms divided by the square of height in meters. Plasma triglycerides and total cholesterol were determined by enzymatic methods, and high-density lipoprotein cholesterol (HDLC), low-density lipoprotein cholesterol (LDLC), apolipoprotein A and apolipoprotein B were determined by homogeneous assays. Fasting plasma glucose was measured by the Glucorder analyzer.

### Genotyping

Genomic DNA was extracted from peripheral blood leukocytes using the TIANamp Blood DNA Kit (Tiangen Biotect [Beijing] Co., LTD). A total of five well-defined polymorphisms in apelin/APJ system were genotyped by the TaqMan assay method, including rs3761581, rs56204867 in *apelin* gene and rs7119375, rs10501367, rs9943582 in *APJ* gene. All polymorphisms were located in the promoter regions of both genes. The Taqman probes and primers were obtained from the Applied Biosystems Assay-by-Design Service. The Universal PCR Master Mix from Applied Biosystems was used in a 5-μl total reaction volume containing 10-ng DNA per reaction. For the sake of accuracy, 100 randomly-selected samples were re-genotyped by TaqMan assay method. The results of two batches were exactly matched.

### Statistical analysis

Continuous data are listed as mean (standard deviation) and compared by *t-test* or Mann-Whitney *U* test where appropriate. Genotype and allele distributions were compared between patients and controls by χ^2^ test or Fisher's exact test where appropriate. Deviation from Hardy-Weinberg equilibrium was evaluated by χ^2^ test in control participants. Linkage disequilibrium of five examined polymorphisms was assessed by Haploview v4.2 (available at the website: http://www.broadinstitute.org/scientific-community/science/programs/medical-and-population-genetics/haploview/downloads). The risk prediction for hypertension was determined by binary Logistic regression analysis under additive and dominant models. The effect-size estimates were denoted as odds ratio (OR) and its 95% confidence interval (95% CI). Multiple comparisons were controlled by the Bonferroni correction. Unless otherwise indicated, a two-tailed *P value* of < 0.05 was accepted as statistical significance.

Allele combination of multiple polymorphisms was derived and its frequency was estimated by the haplo.em program. The risk prediction of derived allele combinations for hypertension was determined by the haplo.cc and haplo.glm programs. The relation between derived allele combinations as a whole and a certain phenotype was delineated by the haplo.score program. The haplo.em, haplo.cc, haplo.glm and haplo.score programs were obtained from the Haplo.stats (v1.4.0) under the “R-project v2.11.1” environment. Simulated *P value*s were based on 1000 replicates. The statistical power was estimated by the EpiCalc (v.2.15.1.0) under the “R-project v2.11.1” environment. Haploview plot of linkage disequilibrium was delineated to measure linkage disequilibrium [28].

## Abbreviations

SBP: systolic blood pressure
DBP: diastolic blood pressure
BMI: body mass index
TG: triglycerides
TC: total cholesterol
HDLC: high-density lipoprotein cholesterol
LDLC: low-density lipoprotein cholesterol
OR: odds ratio
95% CI: 95% confidence interval
ApoA: apolipoprotein A
ApoB: apolipoprotein B.
